# Real-time sentinel lymph node biopsy guidance using combined ultrasound, photoacoustic, fluorescence imaging: *in vivo* proof-of-principle and validation with nodal obstruction

**DOI:** 10.1038/srep45008

**Published:** 2017-03-22

**Authors:** Jeeun Kang, Jin Ho Chang, Sun Mi Kim, Hak Jong Lee, Haemin Kim, Brian C. Wilson, Tai-Kyong Song

**Affiliations:** 1 Department of Electronic Engineering, Sogang University, Seoul, 04107, South Korea; 2 Sogang Institute of Advanced Technology, Sogang University, Seoul, 04107, South Korea; 3 Department of Biomedical Engineering, Sogang University, Seoul, 04107, South Korea; 4 Department of Radiology, Seoul National University of Bundang Hospital, Kyeonggi-do, 13620, South Korea; 5 Princess Margaret Cancer Centre, University Health Network, M5G 1L7, Canada; 6 Department of Medical Biophysics, Faculty of Medicine, University of Toronto, Ontario M5G 1L7, Canada

## Abstract

Precise sentinel lymph node (SLN) identification is crucial not only for accurate diagnosis of micro-metastases at an early stage of cancer progression but also for reducing the number of SLN biopsies (SLNB) to minimize their severe side effects. Furthermore, it is desirable that an SLNB guidance should be as safe as possible in routine clinical use. Although there are currently various SLNB guidance methods for pre-operative or intra-operative assessment, none are ideal. We propose a real-time SLNB guidance method using contrast-enhanced tri-modal images (i.e., ultrasound, photoacoustic, and fluorescence) acquired by a recently developed hand-held tri-modal probe. The major advantage of tri-modal imaging is demonstrated here through an *in vivo* study of the technically-difficult case of nodal obstruction that frequently leads to false-negative results in patients. The results in a tumor model in rabbits and normal controls showed that tri-modal imaging is capable of clearly identifying obstructed SLNs and of indicating their metastatic involvement. Based on these findings, we propose an SLNB protocol to help surgeons take full advantage of the complementary information obtained from tri-modal imaging, including for pre-operative localization, intra-operative biopsy guidance and post-operative analysis.

Sentinel lymph node biopsy (SLNB) is widely used as a minimally-invasive method to determine whether metastasis has occurred in early-stage breast cancer patients. SLNB is usually conducted to select the optimal therapeutic approach, depending on the nodal metastatic status^1^,^2^. SLNB significantly reduces post-operative complications associated with conventional axillary lymph node dissection (ALND)^3^,^4^. Previous studies have reported that up to 30% of breast cancer patients who underwent ALND develop lymphedema. There are additional potential complications such as nerve injury, seroma formation, numbness or limited arm movement^5^. Generally, SLNB comprises the following steps: (1) either exogenous contrast dye or radioactive tracer is administered systemically or around the site of the primary tumor; (2) the operator then identifies the lymph node first reached by the contrast agent, i.e. the so-called sentinel lymph node that has the highest potential for metastasis because of the trapping of tumor cells detaching from the primary tumor; (3) the nodes are resected and (4) frozen-section histopathology is conducted sequentially to determine the cancer stage, which impacts decision-making for further diagnosis and/or treatment. Hence, efficacious pre- and intra-operative SLNB guidance are crucial for successful breast cancer screening and therapy. Ideally, this should precisely localize SLNs and ascertain if there is multiple-basin drainage, while also minimizing the number of invasive procedures required^6^.

There are several SLNB guidance methods currently available, as summarized in Table 1. Several imaging modalities have been used for pre-operative localization and staging of SLNs^7^. Lymphoscintigraphy (LS) is generally recommended before SLNB, since it has high sensitivity to multiple-basin drainage^8^. However, LS has poor spatial resolution (~20 mm) and insufficient precision for SLN localization with a single projected image^9^. Computed tomography (CT) has shown promise for SLN localization, due to its high spatial and temporal resolution. However, it involves risk from the iodinated contrast agent and the high radiation dose^10^. Although positron emission tomography (PET) can detect malignant tumors through their enhanced glycolytic rate, it has poor spatial resolution (1–2 mm) and suffers from potential interference from infection and lymph-node inflammation^11^. Contrast-enhanced magnetic resonance imaging (CE-MRI) can safely provide the morphological and functional information on SLNs with good spatial resolution (50 μm), but identification of metastatic axillary lymph nodes is limited to nodes larger than 5 mm, missing smaller SLNs that may contain early-stage micrometastasis^12^. Furthermore, CE-MRI is an expensive modality and may be not suitable for real-time SLN localization during surgery. As a cost-effective and real-time method, pre-operative contrast-enhanced ultrasound (US) imaging with microbubble contrast agents is a viable alternative. However, its low contrast resolution and high user dependence limit the accuracy of SLN identification^13^. Recently, photoacoustic (PA) imaging has emerged as a promising modality for image-based guidance and is capable of simultaneously providing optical molecular contrast with deep tissue imaging (several cm) and acoustic spatial resolution (~800 μm)^14^,^15^. Preclinical^16^,^17^ and clinical^18^ studies have shown that PA imaging can identify SLNs after injection of near-infrared contrast dyes (e.g., methylene blue and indocyanine green) and can guide biopsy with clear needle visualization. A combined US/PA imaging system for human use has recently become commercially available and a first-in-human study demonstrated the ability of PA imaging to identify metastatic SLNs in melanoma patients, both *ex vivo* and *in vivo*; the results were compared to those from a separate fluorescence (FL) detector used pre- and post-operatively^19^.

There are three representative intra-operative SLNB guidance methods. Visual identification of administered blue dye (BD) is the most popular, because of its intuitive nature and cost-effectiveness. However, it has a low SLN identification ratio (<65%), defined as the ratio of the number of true SLNs to the number of suspicious SLNs, and visibility can be lost in deep-lying SLNs in obese patients^20^,^21^. Radioactivity detection with a gamma-ray probe (RD) is also used to map the SLN basin. RD has an SLN identification ratio of up to 93%, with good depth coverage (>50 mm)^20^. However, the background signals can be high and there is a lack of depth feedback for the detected positive nodes. Additionally, the method is expensive and there is an associated radiation risk^22^. Near-infrared fluorescence detection (FLD) has been recently proposed as an alternative and has the merit of a negative predictive value (>92%) using administered fluorescein dyes, activatable beacons or fluorescent nanoparticles (NPs)^23^. In addition to high contrast, FLD has the distinct advantage of providing wide-field *en face* images that correspond to the surgeon’s view. However, it is necessary to employ a complementary tool for depth profiling, since FLD has a shallow penetration depth of about a few millimeters.

Currently, SLNB is conducted with different guidance methods during the pre- and intra-operative stages. Since all pre-operative methods except US and PA are incapable of real-time imaging, there is a disconnection with pre-operative imaging during surgical procedures, which may reduce the surgical precision. Additionally, all current intra-operative SLNB guidance methods are susceptible to SLN misidentification in technically difficult cases, e.g. SLN basin occlusion and deep-lying SLN basins that result from factors such as remodeling of the lymphatic system (lymphangiogenesis, lymphatic enlargement), nodal invasion by tumor cells and patient obesity^21^,^24^,^25^. In these cases, it is difficult to locate the administered contrast dye in the surgical field-of-view; for example, 59% of palpable SLNs in penile cancer patients did not take up dye due to nodal obstruction and rerouting to other lymph nodes^26^, while 66.7% of the total false-negative cases occurred for the same reasons^27^.

Here we demonstrate that combined US, PA and FL images, using a recently-developed tri-modal probe, can potentially address these issues. There have been a few previous attempts to integrate these three imaging modalities for microscopic and endoscopic applications^28^,^29^. Our approach is that a fully integrated hand-held probe can simultaneously produce *en face* and cross-sectional real-time images of suspicious SLN lesions during both pre- and intra-operative procedures. In particular, this study confirms that tri-modal imaging has the advantage of identifying SLNs in the technically-difficult case of nodal obstruction and of indirectly estimating the likelihood of metastatic SLN involvement. Based on the findings from this initial proof-of-principle study, we propose an SLNB protocol to help surgeons take full advantage of the complementary information from tri-modal imaging in each operative session: pre-operative localization, intra-operative biopsy guidance and post-operative analysis.

## Results

### Tri-modal imaging system

**Figure 1:**
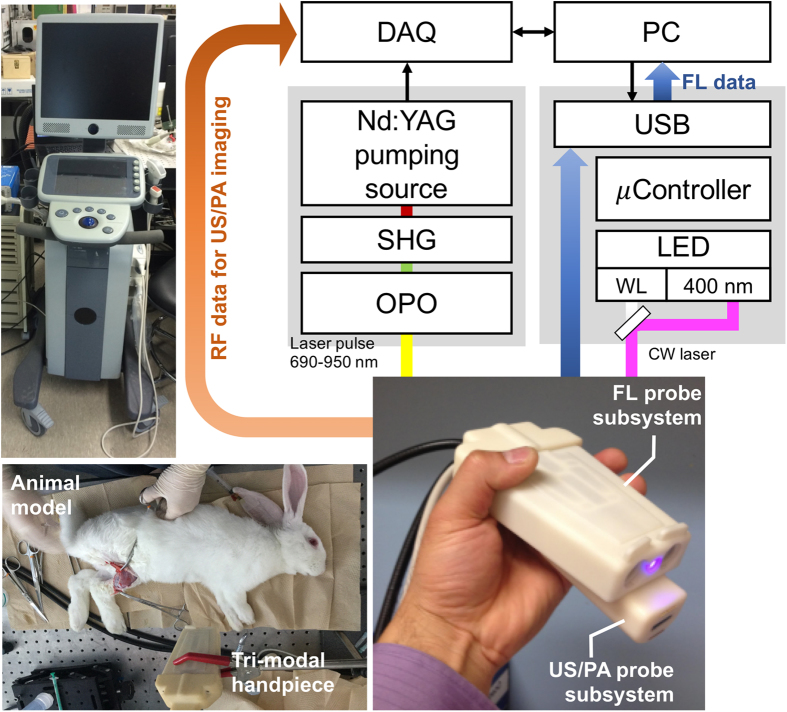
Schematic diagram of the tri-modal imaging system and probe. WL: white light, SHG: second-harmonic generator, OPO: optical parametric oscillator, DAQ: data acquisition board.

Combined US, PA, and FL imaging enables us to obtain, respectively, morphological, functional and molecular information in a single procedure, safely and in real-time. The tri-modal probe shown in Fig. 1 can be used in both pre- and intra-operative assessments. We envisaged that combined US and PA modalities would be used pre-operatively and that FL imaging or all three modalities would then be used intra-operatively. The probe was designed to allow the combined US/PA and FL subsections to be physically separated if required. The combined US and PA images provide cross-sectional depth profiles over several centimeters, whereas the FL (and white-light) images show the *en face* plane across the surgical regions of interest.

### Pre-operative location of SLN enlarged by nodal obstruction

**Figure 2:**
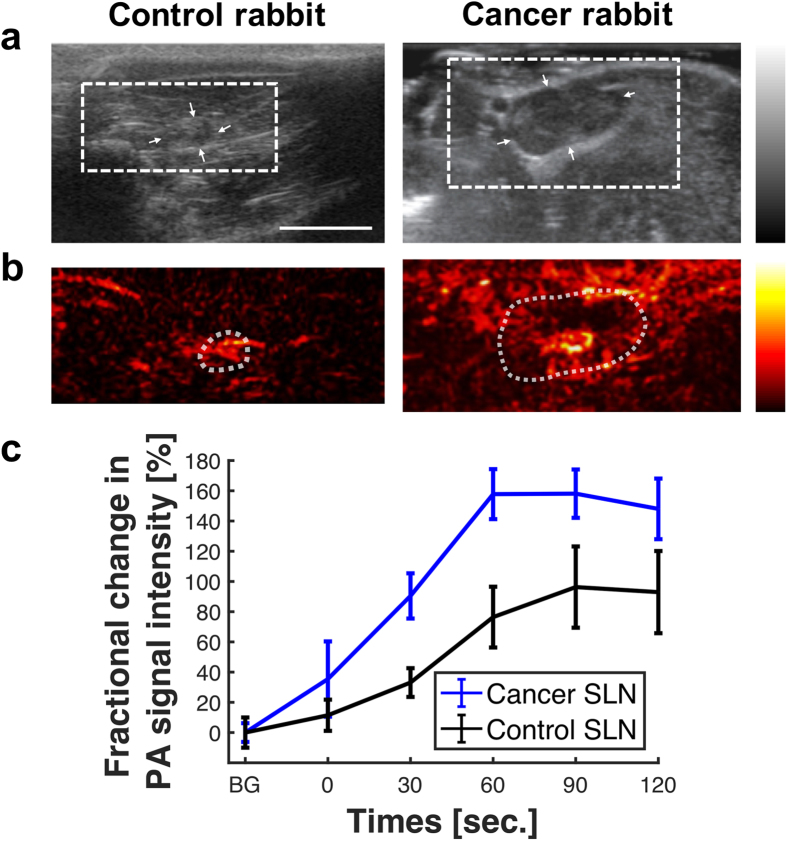
(**a**) US and (**b**) PA cross-sectional *in vivo* images of control (left) and tumor-bearing (right) rabbits acquired in the pre-operative localization session. SLNs are indicated by the white arrows in the US images. The PA images, taken at 90 s after dye injection, covered the region of interest indicated by the dotted rectangle in the US images (see Fig. S1 for the PA images acquired at 0, 30, 60 and 90 s.). The white scale bar in the US image indicates 1 cm. (**c**) PA intensity as a function of time following contrast injection, averaged over the region of interest indicated by white dotted circles in the PA images. BG denotes resting-state background.

In order to illustrate the potential utility of the tri-modal imaging, US imaging was used first to identify a suspicious SLN located near the primary tumor in the inguinal region of a rabbit tumor model as shown in Fig. 2(a). This showed that the SLN was enlarged compared to normal nodes: 7 mm × 14 mm *versus* 4 mm × 5 mm. This suggested tumor spread into the SLN, which caused nodal obstruction. The suspicious SLN was confirmed by PA images acquired after administering a multi-modal contrast dye into the primary tumor. The same dye was also injected into the tissue surrounding a lymph node in the case of a non-tumor-bearing rabbit. The PA images were acquired once every 30 s for 10 min following contrast administration: Fig. 2(b). The most marked change in PA intensity occurred at 60 s in both control and cancer models, as shown in Fig. 2(c). The rate of PA intensity increase was higher in the cancer model (2.62-fold increase over baseline per second) than in the normal case (1.27 per second), while PA intensity increased by up to 60% in the cancer model than in the control. These findings may be due to the increased permeability of the contrast agent into the metastatic node as a result of lymphangiogenesis and lymphatic enlargement^24^. Additionally, the increased PA intensity due to the contrast agent within the metastatic SLN may be the result of the blockage of flow to the efferent lymph vessel, as verified by post-operative *ex vivo* FL images and H&E histology (see below).

### Intra-operative biopsy guidance and post-operative analysis

**Figure 3:**
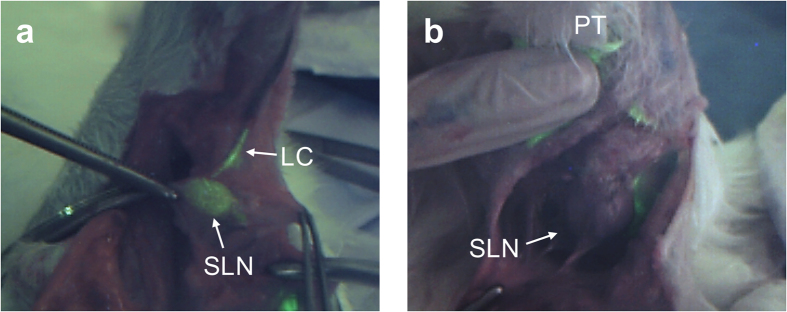
FL images obtained during the intra-operative guidance session: (**a**) control and (**b**) tumor-bearing; PT-primary tumor, LC-lymphatic channel.

After pre-operative localization by US/PA imaging, SLNB was conducted with intra-operative FL imaging guidance. The inguinal region of interest as localized by the US and PA images was surgically opened to facilitate this. After opening, FL imaging was performed to confirm the lymph node for biopsy. As shown in Fig. 3(a), the SLN and lymphatic channel of the control rabbit were clearly observed in the FL image through the marked contrast between the target and other lymph nodes, enabling accurate resection of the SLN. However, there was no marked contrast of the SLN in the corresponding FL image obtained in the tumor-bearing rabbit, thus hindering intraoperative identification of the SLN, unless SLN enlargement made visual identification possible and the PA image was available. This inconsistency between the PA and FL observations in the metastatic SLN occurred mainly because the *en face* FL images have only 1–2 mm depth sensitivity. Hence, significant FL signal can only be detected if the node is well perfused by the fluorescent agent close to the SLN surface. On the other hand, PA imaging provides a cross-sectional image with imaging depth of about several centimeters.

**Figure 4:**
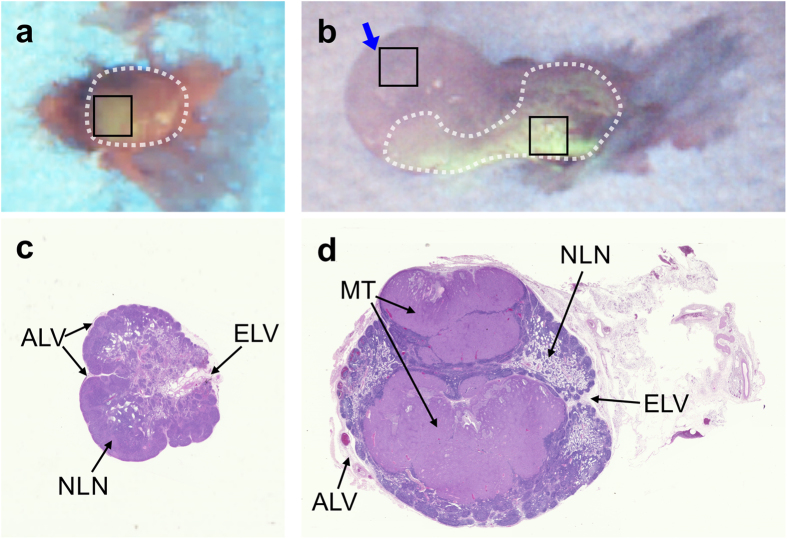
Post-operative analysis: *ex vivo* FL validation of the resected SLNs from (**a**) control and (**b**) tumor-bearing rabbits. The blue arrow in (**b**) indicates the direction of FL imaging available during *in vivo* surgical guidance. The black rectangles indicate the regions-of-interest for FL intensity measurements. H&E stained sections are shown for the corresponding (**c**) control and (**d**) tumor-bearing SLN: MT-metastatic tumor, EL-efferent lymph vessel, ALV-afferent lymph vessel, NLN-normal lymph node tissue.

The nodal obstruction occurring in the SLN of the tumor-bearing rabbit was confirmed by post-operative analysis that included *ex vivo* FL imaging and frozen-section histology. Figures 4(a) and (b) show the distribution of multi-modal contrast dye in the SLN samples from the control and tumor models, respectively. There was enhanced FL contrast in the entire SLN sample from the control rabbit and this agreed well with the intra-operative FL images. However, the FL image of the SLN in the tumor model showed contrast dye accumulating at high concentration only in the posterior aspect of the node, which accounts for the negative intra-operative FL images; the FL intensity was augmented in the posterior side of the SLN sample from the tumor model (216.52 ± 6.54) more than in the control (113.44 ± 0.56), whereas the anterior side of the SLN sample from the tumor model (indicated by the blue arrow in Fig. 4(b)) had a very low FL intensity (94.75 ± 1.07). This result agrees well with the pre-operative PA measurement, implying that there was dye accumulation in the SLN basin, likely due to blockage of the contrast agent flow to the efferent lymph vessel caused by the nodal obstruction. This was confirmed by H&E histology that showed marked nodal obstruction in the tumor-bearing model, as shown in Fig. 4(c) and (d). Additionally, it was clear that lymphatic flow to the efferent lymphatic vessel was substantially blocked, confirming the main mechanism for the localized concentration of contrast agent in the PA imaging: Fig. 2(b).

## Discussion

According to a 2016 estimation by Siegel *et al*., breast cancer comprises some 29% of total cancer diagnoses in the United States and is the second leading cause of cancer-related deaths in women after lung cancer (14% of total cancer-related deaths)^30^. While treatment of breast cancer continues to advance, early detection is still the best defense. In this context, fine needle aspiration biopsy (FNAB) under US imaging guidance serves as a minimally-invasive screening tool. Recently, it has been shown that combined US and PA imaging can clearly identify a biopsy needle and SLNs, so improving the accuracy of FNAB. However, FNAB has a high false-negative rate of 11–20% and highly variable sample rate (0–53%)^31^. Hence, SLNB is needed to confirm LN metastasis. A major challenge in SLNB is that there is no guidance method that meets all the requirements for precise pre-operative localization and intra-operative biopsy guidance, in real time and with minimal risk. We have demonstrated in this initial preclinical study that a tri-modal approach integrating US, PA and FL imaging may provide a potential solution. In particular, we have shown, at least in this animal model, that the approach can identify SLNs in technically difficult cases such as nodal obstruction. A suspicious SLN can be identified non-invasively in real-time by the dynamic changes in the PA image intensity, while its morphology is obtained from the corresponding US images. Both tumor-bearing and control models showed clear PA contrast enhancement within 10 min after multi-modal contrast dye administration. This would be highly informative to help localize the suspicious foci pre-operatively, thereby reducing the required biopsy incision length. Intra-operatively, the *en face* field-of-view of real-time FL imaging can clearly delineate the target SLN to be resected, which could enhance clinical outcomes such as improved sensitivity and SLN identification ratio. As shown in Fig. 3(a), lymphatic drainage through the connected lymph channel was clearly observed in the FL image of the control rabbit model. Although FL imaging failed to reveal the heavily metastatic SLN in the cancer model (Fig. 3(b)), the complementary information from the cross-sectional PA imaging facilitated precise excision of the target SLN. FL imaging and H&E histology confirmed that the excised SLN was obstructed in a manner that frequently causes a false-negative result. This discrepancy between PA and FL imaging may be used to advantage to determine indirectly the metastatic status of SLNs during resection. Furthermore, this may lead to development of an indirect method for pre-operative diagnosis through determination of the correlation between the PA signal distribution in a SLN and its metastatic status.

The proposed tri-modal approach may also improve the effectiveness and productiveness of SLNB. Combined US and PA imaging is useful to localize suspicious foci pre-operatively but is inefficient and time-consuming for intra-operative use, since both modalities require contact of the probe with the tissue surface using coupling gel and the images are cross-sectional in nature and requires manual scanning of the probe. By contrast, FL imaging enables the surgeon to quickly examine the entire surgical region for intra-operative SLN identification due to its *en face* field of view and non-contact imaging. Only in the case where FL imaging fails to reveal a heavily metastatic SLN are combined intraoperative US and PA necessary. The tri-modal system is particularly suited to this task since it allows rapid mode switching.

**Figure 5:**
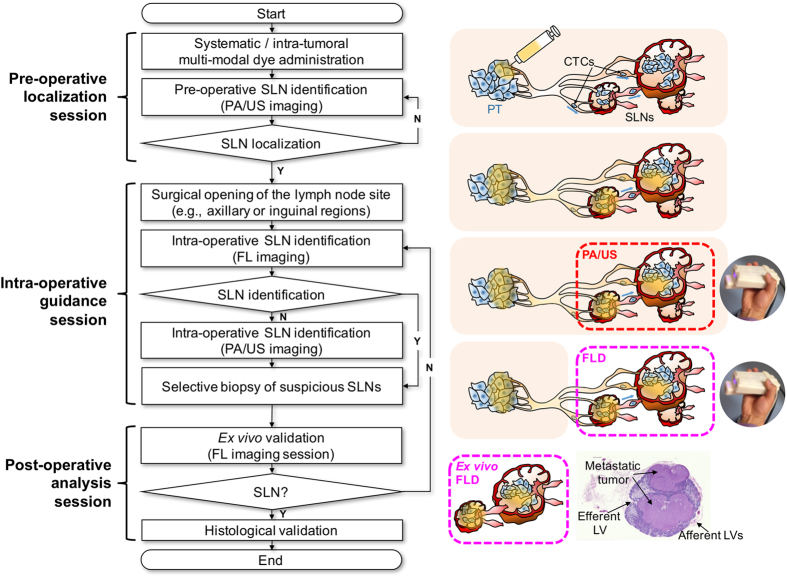
Flowchart of proposed SLNB guidance using contrast-enhanced tri-modal imaging.

Based on our *in vivo* experimental results, we propose a novel clinical protocol using the contrast-enhanced tri-modal SLNB guidance, as summarized in Fig. 5, comprising three sequential sessions: pre-operative localization, intra-operative guidance, and post-operative analysis. At each stage, the real-time and molecular imaging capabilities of tri-modal imaging are fully utilized such that both effectiveness and accuracy of the SLNB procedures are improved. Thus, the protocol starts with delivery of contrast agent, either systemically or into the local lymphatic system around the tumor. The contrast agent may be truly tri-modal such as porphyrin-lipid microbubbles^32^, dual-modal contrast agent^33^ or a mixture of PA contrast agent and FL dye^34^. The contrast agents perfuse through the tissue and are then drained into the lymphatic flow, leading to accumulation in SLN basins. In the pre-operative session, accurate localization of these basins is performed with real-time US and PA imaging, providing localization that can be used in subsequent incision and SLNB procedures. The most important objectives at this session include delineation of the morphologic features of suspicious SLNs (e.g., position, size and shape) by using US imaging, and confirmation of multiple basins by observing the PA signal distribution in suspicious SLNs and their change over time. Since combined US and PA imaging allows these observations to be done in a non-invasive manner even for deep-lying SLNs (several centimeters), the surgeon can be confident about the expected surgical outcomes (e.g., SLN depth from the skin surface and number of SLNs to be biopsied). In the intra-operative guidance session, the surgeon first approaches the SLNs by opening the selected region of interest with the closest and shortest incision length. The target SLNs can then be identified in the FL image. If no significant FL emission is detected from the target SLNs, then PA imaging is used to determine if the SLNs are technically difficult to approach. Thereby, the surgeon can precisely extract the most suspicious SLNs regardless of their metastatic state. The subsequent post-operative analysis session consists of *ex vivo* FL imaging using the tri-modal probe and frozen-section histological validation. Immediately after SLNB, real time FL imaging indicates if the extracted SLNs are drained from the entry point of the administered contrast agent. This enables the surgeon to match the information quickly and efficiently from the pre-operative session in terms of the size and degree of contrast agent accumulation. This process provides important feedback to the surgeon, who can immediately continue resection to find another suspicious focus in the case of inconsistent information. The frozen-section histological analysis is the final procedure to confirm whether the SLN is metastatically involved, allowing the final treatment decision based on the stage of progression of the tumor. This proposed protocol can be realized with minimal change of the current SLNB workflow, in which US and FL imaging are used separately either pre- or intra-operatively. Another advantage is that it avoids the complications due to radionuclides.

In this SLNB guidance, the use of a tri-modal contrast agent fully leverages the advantages of the combined US, PA and FL imaging. In the present proof-of-principle study, we used mixed contrast agent comprising methylene blue and fluorescein. However, this simple approach gives sub-optimal PA and FL contrast and prevents full utilization of the US imaging. Recently developed novel tri-modal contrast agents such as porphyrin-shell microbubbles should address this problem^32^. The use of these novel microbubbles can be further enhanced by applying ultrasound conversion to corresponding nanoparticles in order to complement the intrinsic contrast due to enhanced permeability and retention (EPR) effect^35^. This approach requires some technical changes to the FL subsystem of the tri-modal instrument, in particular switching of the FL excitation light source (400 ± 10 nm) and 450 nm long-pass filter to the near-infrared spectral range to match the fluorophore spectra^32^, thereby improving the signal-to-noise and enabling deeper imaging depth or the use of established contrast agents such as indocyanine green: 780 nm and 825 nm for peak spectral absorbance and emission. Furthermore, the development of robust multi-spectral PA imaging to differentiate the signal of the administered contrast agent flowing into the SLN from the surrounding blood background should facilitate quantitative estimation of nodal obstruction prior to surgical incision. In order to assess their clinical utility, the range of validation studies should be extended in terms of the number of cases at different stages of metastatic progression.

## Methods

### Tri-modal imaging system

The tri-modal imaging system consists of an integrated, hand-held tri-modal probe, together with a combined US and PA sub-system and a separate FL imaging sub-system, as shown in Fig. 1. The tri-modal probe has two subsections: a combined US/PA part and a FL part that can be physically separated depending on the required mode of operation. For both US and PA imaging, the combined US/PA probe was connected to a modified commercial US imaging scanner with a research package (SonixTouch and SonixDAQ, Ultrasonix Medical Corp., Canada). To induce the PA signals, pulsed laser light was generated by a mobile, second-harmonic (532 nm) Nd:YAG laser pumping an optical parametric oscillator (OPO) system (Hanbit Laser Corp., Korea). The tunable range is 690–900 nm and the maximum pulse repetition frequency is 20 Hz. Light was delivered into the probe through bifurcated fiberoptic bundles, each 40 mm long and 0.88 mm wide, giving a laser illumination area of 70.4 mm^2^. In the present study, a wavelength of 700 nm was used for PA imaging, at which the pulsed laser system was stable enough to accurately track the PA intensity over time. The total pulsed laser energy injected into the 9.5-mm fiberoptic bundle was 40 mJ per pulse. The delivered energy density from the contact probe was then 12 mJ/cm^2^ (8.5 mJ/70 mm^2^) on the skin surface, which is below the ANSI safety limit of 20 mJ/cm^2^ in the near-infrared wavelength range. In US/PA mode, a 32-frame data set was obtained every 30 s at a frame rate of 10 Hz to prevent potential photobleaching of the contrast dye. The FL imaging system provided *en face* images after delivering/collecting the light to/from the probe. In the intra- and post-operative FL sessions, FL images were acquired in 6–14 ms, yielding 4.6–31.4 frames/s via the custom-made acquisition software. The incident power was 100 mW at a working distance of 15–20 cm. No signal decrease was observed due to photobleaching. Detailed technical and performance information can be found in Kang *et al*.^36^ and Figs S3 and 4 in the Supplementary Information give the sensitivity and reliability test results.

### Image acquisition and reconstruction

The US images were acquired using the default clinical settings of the US imaging system (SonixTouch and SonixDAQ). For the PA image reconstruction, 32 frames of pre-beamformed RF data were consecutively acquired and averaged to enhance the signal-to-noise ratio and then unfiltered back projection beamformation was conducted. DC rejection, envelope detection and digital scan conversion (DSC) were subsequently performed. A 128-tap low-pass filter (LPF) with a cutoff frequency of 1 MHz was used for DC rejection, while envelope detection used Hilbert transform followed by band-pass filtering from 2 MHz to 8 MHz. The FL images were displayed in true color with neither gain nor RGB color balance adjustment applied to the raw CMOS cameras pixels.

### Multi-modal contrast agent preparation

A mixture of methylene blue and fluorescein (M9140 and F6377: Sigma-Aldrich Co., St. Louis, MO, USA) was used as a multi-modal contrast agent; their optical characteristics are presented in the Supplementary Information, each being well known for PA and FL imaging contrast dyes, respectively. They were mixed at equal weight (2.5 mg) in 5 mL distilled water, giving molar concentrations of 1.56 and 1.32 mM, respectively: see the Supplementary Information for the justification of determining the molar concentrations.

### Animal preparation

All animal experiments in conjunction with the imaging methods were conducted in accordance with the guidelines and regulations approved by the Institutional Review Board of Seoul National University Bundang Hospital, South Korea (BA1407-157/037-01). As described in more detail in the Supplementary Information, cancerous SLNs were generated in one adult male New Zealand White rabbit (2 kg) by intramuscular injection of 1 cc of VX2 tumor cell suspension into the thigh. Metastatic progression to SLNs was confirmed through US imaging of the inguinal region at 2 or 3 weeks later. The tumor-involved SLNs grew to approximately 15 mm diameter, which was about twice the size of normal SLNs in the control non-tumor-bearing rabbit. The *in vivo* experiments were conducted after 19 days following tumor induction, at which 5 mL (2.5 mL/kg) of contrast agent was administered *via* peri-tumoral injection under gas-inhalation anesthesia (Isoflurane at 4–5% and 1–2% for induction and maintenance, respectively, with 100% oxygen). Body temperature was maintained on an electrical heating pad. After imaging, positive SLNs identified were resected for *ex vivo* FL validation and H&E stained histology. The rabbits were sacrificed by intravenous injection of potassium chloride.

## Additional Information

**How to cite this article:** Kang, J. *et al*. Real-time sentinel lymph node biopsy guidance using combined ultrasound, photoacoustic, fluorescence imaging: *in vivo* proof-of-principle and validation with nodal obstruction. *Sci. Rep.* **7**, 45008; doi: 10.1038/srep45008 (2017).

**Publisher's note:** Springer Nature remains neutral with regard to jurisdictional claims in published maps and institutional affiliations.

## Supplementary Material

Supplementary Information

## Figures and Tables

**Table 1:** Conventional pre- and intra-operative SLNB guidance methods.

Aims	Pre-operative						Intra-operative		
	Precise SLN localization overview around the suspicious nodal region (axillary or inguinal regions) detection of multiple-basin drainage						Precise SLN localization with wide field-of-view in real-time		
Modality	LS	CT	PET	MRI	US	PA	BD	RD	FLD
Spatial resolution	20 mm	50 μm	1–2 mm	50 μm	400 μm	800 μm	Unaided visual resolution	>10 mm	100 μm
Throughput	Low				High				
Image format	Projection in single direction	Tomography			Cross-section and/or coronal planes		*En face* to the tissue surface	Freehand point scanning	*En face* to the tissue surface
Sensing depth	>30 cm				>20 cm	~7 cm	~1 mm	>50 mm	~1–2 mm
Exogenous contrast agent	Radio-active tracer	Iodinated contrast agent	Positron-emitting radionuclide tracer	Magnetic contrast agent (e.g., paramagnetic iron oxide, gadolinium chelates)	Micro-bubbles	Molecular dyes or NPs with high optical absorption	Methylene blue	Radio-active tracer	Molecular dyes, activatable beacons, NPs
Cost	High	High	High	High	Low	not established	Low	High	Medium
Ionization radiation?	yes	yes	yes	no	no	no	no	no	no
