# Supplementary material for: Real-time sentinel lymph node biopsy guidance using combined ultrasound, photoacoustic, fluorescence imaging: in vivo proof-of-principle and validation with nodal obstruction
Source: Sci Rep. 2017 Mar 22;7:45008. doi: 10.1038/srep45008 (PMC5361205; doi:10.1038/srep45008)
Supplement: Supplementary Information [file srep45008-s1.doc]

Supplementary Information

**Real-time sentinel lymph node biopsy guidance using combined ultrasound, photoacoustic, fluorescence imaging: *in vivo* proof-of-principle and validation with nodal obstruction**

**Jeeun Kang^1^, Jin Ho Chang^1,2,3^*, Sun Mi Kim^4^, Hak Jong Lee^4^, Haemin Kim^3^, Brian C. Wilson^4,5^, and Tai-Kyong Song^1^***

^1^Department of Electronic Engineering, Sogang University, Seoul, 04107, South Korea

^2^Sogang Institute of Advanced Technology, Sogang University, Seoul, 04107, South Korea

^3^Department of Biomedical Engineering, Sogang University, Seoul 04107, South Korea

^4^Department of Radiology, Seoul National University of Bundang Hospital, Kyeonggi-do, 13620, South Korea

^4^Princess Margaret Cancer Centre, University Health Network, M5G 1L7, Canada

^5^Department of Medical Biophysics, Faculty of Medicine, University of Toronto, Ontario M5G 1L7, Canada.

*[jhchang@sogang.ac.kr](mailto:jhchang@sogang.ac.kr); [tksong@sogang.ac.kr](mailto:tksong@sogang.ac.kr);


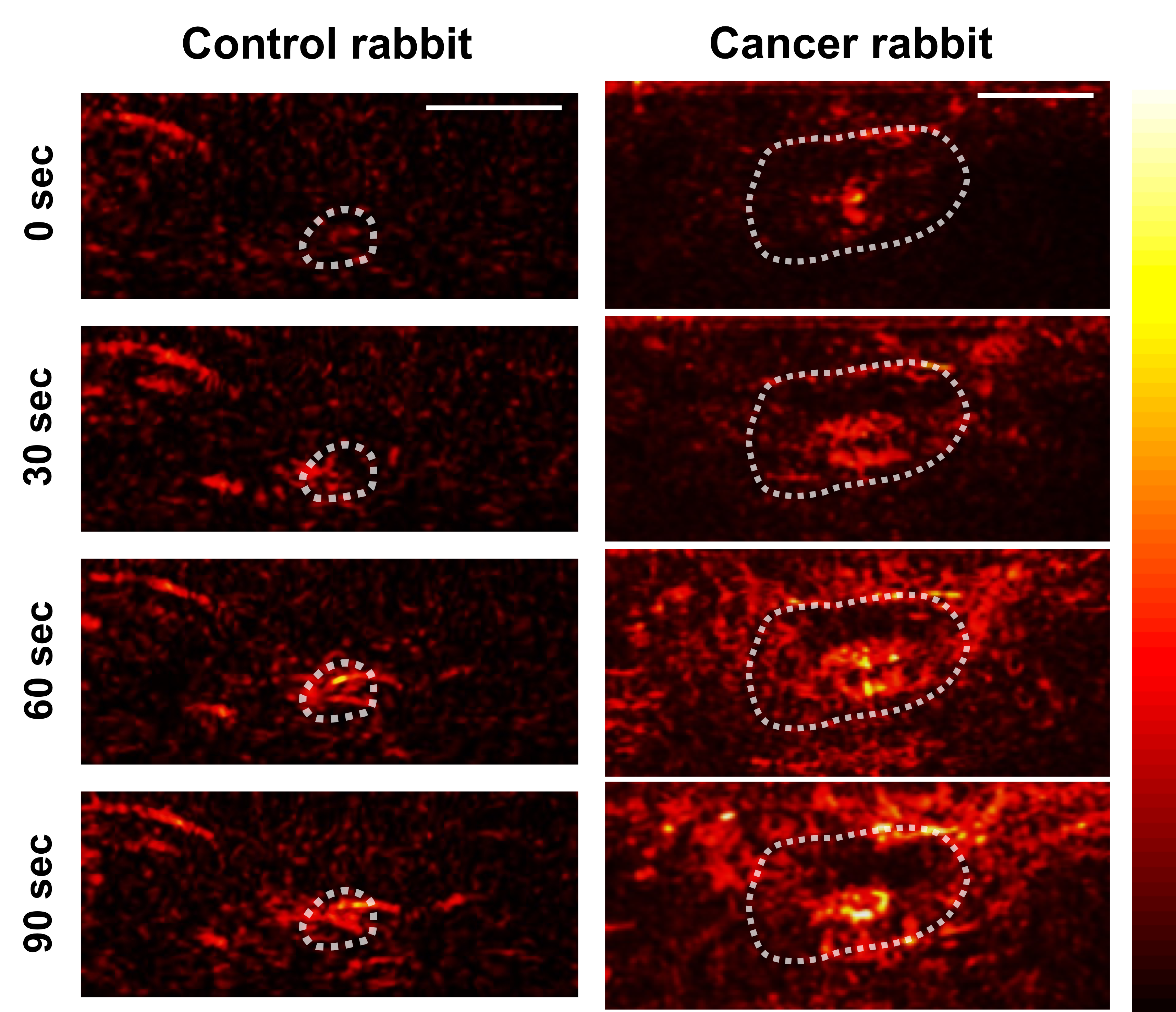


**Figure S1.** PA cross-sectional in vivo images of control (left) and tumor-bearing (right) rabbits acquired in the pre-operative localization session over a period of time (0 – 90 sec). The white bars indicate 10 mm and the white dotted circles represent the region of interest (i.e., the suspicious SLN).


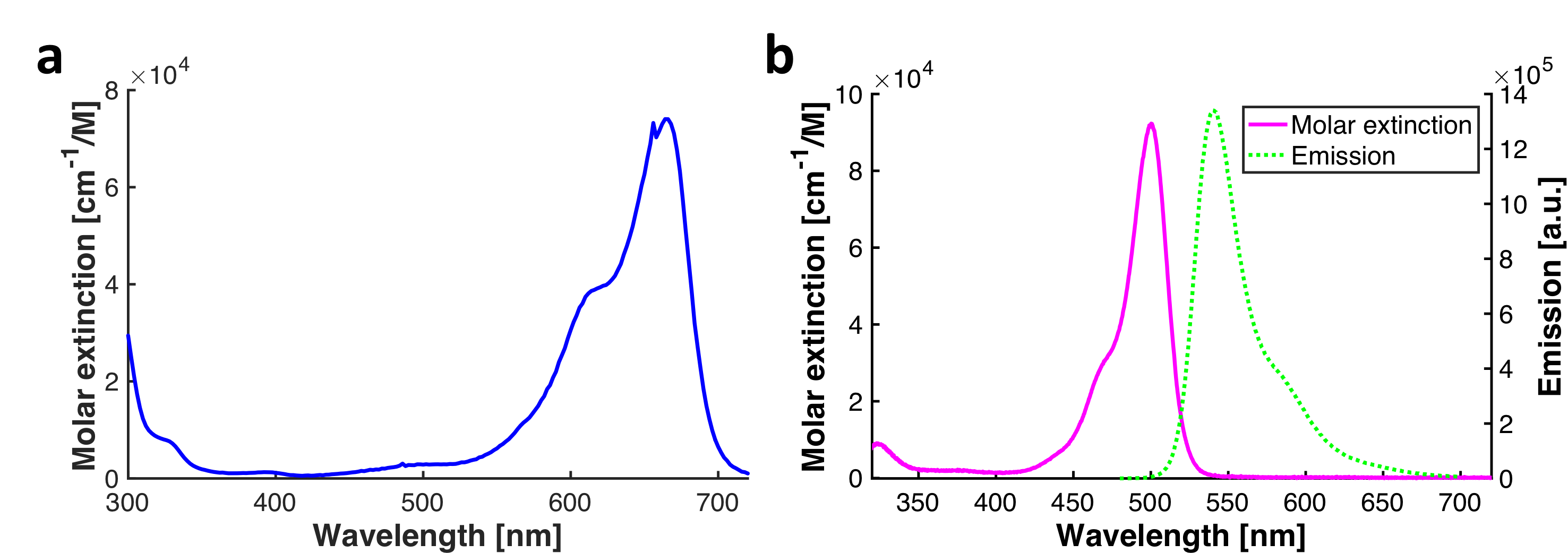


**Figure S2.** Optical characteristics of the contrast dyes: (a) molar extinction coefficient of methylene blue and (b) molar extinction and emission spectrum of fluorescein in basic ethanol.

**Justification of molar concentrations of multi-modal contrast agent.** The concentration of methylene blue was determined based on the fact that a methylene blue of 0.5 mM produced a PA signal-to-noise ratio (SNR) of 25 dB when it is placed at 2.3 cm depth in chicken breast tissue.^1^ Based on the result, we determined a methylene blue concentration to be 1.56 mM because we expected at least tens of dB in SNR after being diluted in a lymphatic node; the SNRs were 13.30 dB and 18.30 dB in the cases of the control and cancer SLNs at 90 sec after injection. Also, the concentration of fluorescein was determined based on the results of the previous *in vivo* experiment for fluorescence SLNB guidance; a fluorescein solution of 10% for human (i.e., 53 mM) was used.^2^ We divided the molar concentration by the ratio between the total blood volumes of a human and the rabbit used in our experiments (i.e., 5000 ml vs. 114 ml^3,4^). By doing so, a molar concentration of fluorescein for the rabbit model was estimated to be 1.21 mM and we used 1.32 mM in our *in vivo* rabbit experiments.

**Sensitivity test of a tri-modal imaging system.** We conducted phantom experiments to measure the noise-equivalent molar concentration of the two contrast agents (i.e., methylene blue and fluorescein). For PA imaging, a transparent tube (AAQ04133, Tygon®, Saint-Gobain Corp., Courbevoie, France) filled with methylene blue was immersed in a water tank. Different molar concentrations (i.e., 0.5, 1, 2, and 4 mM) were used in the experiment, and the background noise without any absorber targets was also measured. From the experiment, it was seen that the noise-equivalent molar concentration of methylene blue was 179 µM. Note that in the experiment we used the same parameters for both averaging and image reconstruction procedures employed in the *in vivo* experiments. For FL imaging, fluorescein samples with specific molar concentrations of 1, 2, and 4 µM were dissipated in a transparent petri dish. FL intensity was measured with 15 cm working distance, 6 ms exposure time, 100 mW light power, 0 dB gain. A noise-equivalent molar concentration of 718.3 nM was measured for FL imaging.

**Figure S3.** Sensitivity test of the tri-modal imaging system: (a) PA imaging with methylene blue and (b) FL imaging with fluorescein

**Reliability test of a tri-modal imaging system.** For the technical validation of the tri-modal imaging system proposed in this paper, reliability studies for PA and FL imaging were conducted using phantoms for 3 days. For PA imaging, 1.56 mM (2.5mg/5ml) methylene blue filled a transparent tube immersed in a 20% whole milk solution to mimic light scattering of soft tissue.^5^ The distance between the US/PA probe part and tube was controlled to be 7.5, 15.0, and 22.5 mm, respectively. Identical parameters for the data acquisition and reconstruction to the *in vivo* experiments were utilized: 12 mJ/cm^2^ energy density, 32 frame averaging, DC rejection with a 1-MHz cutoff frequency, bandpass filtering from 2-MHz to 8MHz. The mean and standard deviation were calculated among the PA intensities measured during 3 subsequent days. Also, the test for FL imaging was performed during 3 subsequent days for a 5 ml fluorescein solution on a flat, shallow plate. The molar concentration of a fluorescein solution was 1.32 mM (2.5mg/5ml) equal to the value used in the *in vivo* experiments. For the reliability test, the parameters of 15-cm working distance from the FL imaging part, 100mW light power, and 7 ms exposure time were used.

**
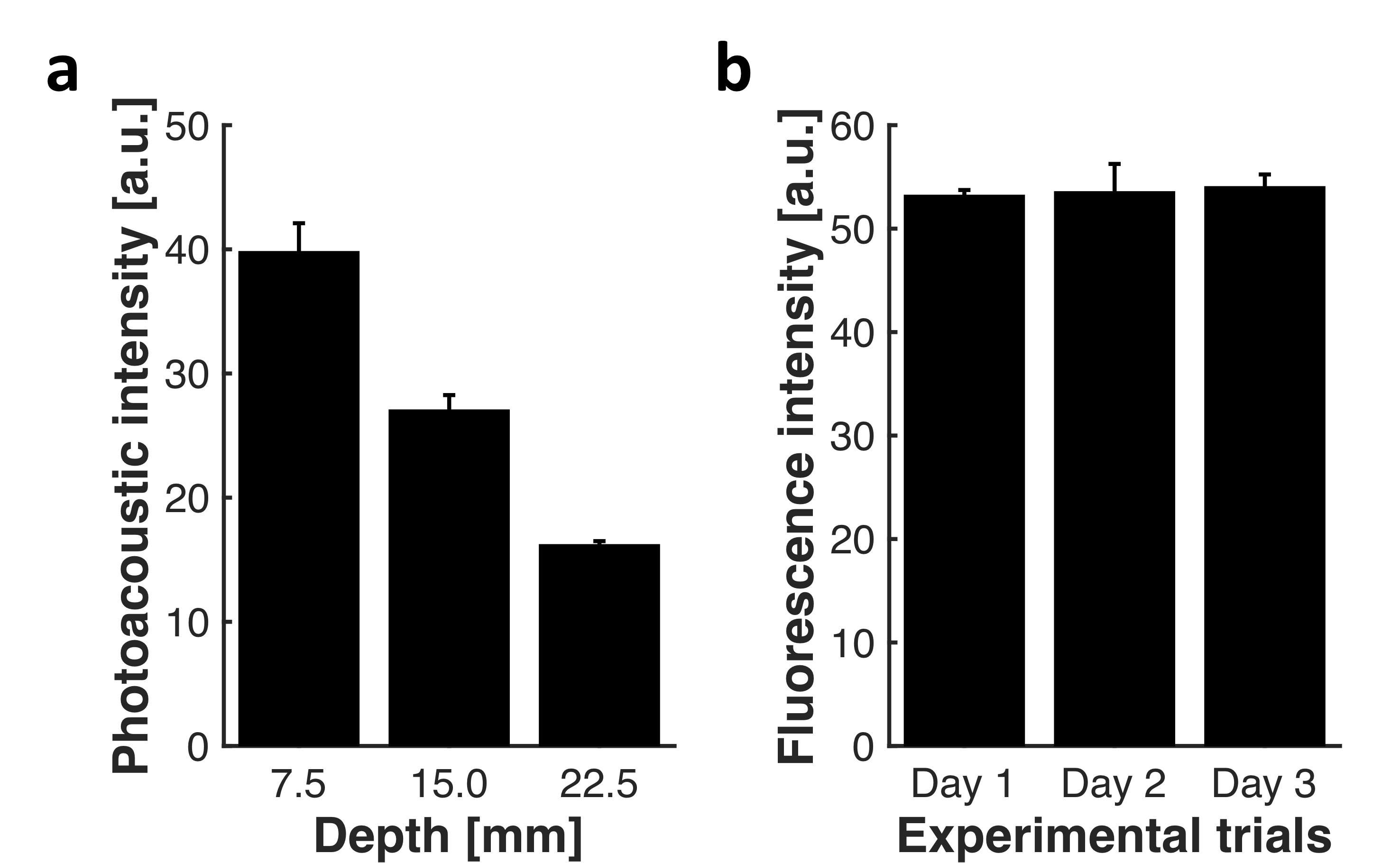
**

**Figure S4.** System stability over 3 subsequent days: (a) mean and standard deviation of PA intensities measured during 3 subsequent days at 7.5, 15, and 22.5 mm, respectively and (b) FL intensities at first, second, and third days.

**Cancer SLN model preparation.** Firstly, a host VX2 tumor was cultivated in the thigh region of a rabbit.^6^ The host VX2 tumor was peeled and separated into multiple 1 x 1 mm^2^ tumor samples using a surgical scissor. The VX2 tumor suspension was produced by compounding the tumor samples into a 5-cc saline solution. To produce a cancer SLN model with nodal obstruction, the VX2 tumor suspension was intra-muscularly injected on a thigh region of another rabbit with 1-cc quantity. The injection was conducted once per rabbit. The metastatic progress of a SLN with nodal obstruction was checked weekly by US imaging; 2-3 weeks later of VX2 tumor suspension injection, the cancer SLN model grew to approximately 15 mm in diameter, about twice the size of normal SLNs in the control non-tumor-bearing rabbit (i.e., 7 mm).

Reference

1. Kim, C., Erpelding, T. N., Jankovic, L. & Wang, L. V. Performance benchmarks of an array-based hand-held photoacoustic probe adapted from a clinical ultrasound system for non-invasive sentinel lymph node imaging. *Philosophical Transactions of the Royal Society A: Mathematical, Physical and Engineering Sciences* **369,** 4644–4650 (2011).

2. Dan, A. G. *et al.* 1% Lymphazurin vs 10% Fluorescein for Sentinel Node Mapping in Colorectal Tumors. *Arch Surg* **139,** 1180–1184 (2004).

3. Morgan, G. E., Mikhail, M. S., Murray, M. J. & Kleinman, W. *Clinical anesthesiology*. (2002).

4. Wachington, I. M. & Hoosier, G. V. in *The Laboratory Rabbit, Guinea Pig, Hamster, and Other Rodents* 57–116 (Elsevier, 2012). doi:10.1016/B978-0-12-380920-9.00001-8

5. Rajian, J. R., Carson, P. L. & Wang, X. Quantitative photoacoustic measurement of tissue optical absorption spectrum aided by an optical contrast agent. *Opt. Express* **17,** 4879–4889 (2009).

6. Kreuter, K. A. *et al.* Development of a rabbit pleural cancer model by using VX2 tumors. *Comp. Med.* **58,** 287–293 (2008).
